# Laparoscopic Hand-Assisted Donor Nephrectomy: A Decade of Single-Center Experience and Outcomes

**DOI:** 10.5152/tud.2025.24161

**Published:** 2025-04-04

**Authors:** Sevim Nuran Kuşlu Çiçek, Amil Huseynov

**Affiliations:** 1 Department of General Surgery, Biruni University, Istanbul, Türkiye; 2 Department of Transplantation, Medicana Hospital, Istanbul, Türkiye; 3 Beykoz University Faculty of Medicine, Istanbul, Türkiye

**Keywords:** Hand-assisted laparoscopic surgery, kidney transplantation, living donor nephrectomy

## Abstract

**Objective:**

Kidney transplantation is the most effective treatment for end-stage renal disease, but the shortage of cadaveric donors has increased reliance on living donors. Traditional open donor nephrectomy, while effective, is associated with significant morbidity. Hand-assisted laparoscopic donor nephrectomy (HALDN) combines the advantages of minimally invasive surgery with the tactile feedback of open surgery. This study presents the outcomes of HALDN procedures performed at the center.

**Methods:**

A total of 1221 living donor nephrectomies performed between September 2009 and August 2021 at Medicana İstanbul Hospital were analyzed. Donor characteristics, surgical details, and postoperative outcomes were recorded. Data analysis was conducted using SPSS version 22.0, with continuous variables assessed for normality and expressed accordingly.

**Results:**

Donor ages ranged from 19 to 87 years (mean 48.50 ± 12.75 years), with 54.8% female donors. The average body mass index (BMI) was 27.99 ± 3.7 kg/m^2^. Left-sided nephrectomies constituted 78.5% of cases, with HALDN performed in 94.7% of these surgeries. Warm ischemia time averaged 99.21 ± 56.67 seconds. The average blood loss was 70 mL, and the mean hospital stay was 4.12 ± 1.2 days. Complications included conversion to open surgery due to bleeding in 3.3% of cases, postoperative atelectasis (0.6%), incisional hernia (0.33%), wound infection (0.16%), and scrotal swelling (0.25%). The findings indicate that HALDN is a safe and effective method for donor nephrectomy, aligning with similar studies regarding operation and warm ischemia times. The minimally invasive nature of HALDN contributes to shorter hospital stays and quicker postoperative recovery. The rate of conversion to open surgery was within acceptable limits, and complications were manageable. Obesity (BMI > 30 kg/m^2^) was identified as a risk factor for incisional hernia, suggesting the need for careful surgical technique in this group.

**Conclusion:**

Hand-assisted laparoscopic donor nephrectomy offers a minimally invasive, safe, and effective alternative for living donor nephrectomy, enhancing donor recovery and potentially encouraging organ donation. Its adoption may play a significant role in reducing the number of patients awaiting organ transplants.

**Main Points**

- Over a 12-year period, 1221 HALDN procedures were performed, demonstrating that HALDN is a safe and effective method for living donor nephrectomy.
- Hand-assisted laparoscopic donor nephrectomy provides benefits of minimally invasive surgery, including shorter hospital stays (mean 4.12 days), reduced postoperative pain, and faster recovery times.
- Complication rates were low and manageable; conversion to open surgery due to bleeding occurred in 3.3% of cases, and obesity (BMI > 30 kg/m^2^) was identified as a risk factor for incisional hernia.
- The adoption of HALDN may encourage organ donation by enhancing donor recovery, potentially reducing the number of patients awaiting organ transplants.

## Introduction

Kidney transplantation is considered the most effective treatment for end-stage renal disease, offering improved quality of life and survival compared to dialysis.^1^,^2^ However, the shortage of cadaveric donors and the increasing number of patients on organ waiting lists have necessitated the procurement of organs from living donors.^3^,^4^ Traditional open donor nephrectomy has been associated with low donor mortality and excellent graft function outcomes.^5^ Nevertheless, it poses significant morbidities for donors, including prolonged hospital stays, postoperative pain, wound complications, and delayed return to normal activities.^6^,^7^ To minimize these drawbacks, laparoscopic donor nephrectomy (LDN) has emerged as a less invasive alternative, providing benefits such as reduced postoperative pain, shorter hospital stay, and faster recovery.^8^,^9^ Laparoscopic donor nephrectomy was first performed by Ratner et al^10^ in 1995, marking a significant advancement in donor nephrectomy techniques. Subsequently, in 1998, Wolf et al^11^ introduced the hand-assisted laparoscopic donor nephrectomy (HALDN) technique, combining the advantages of laparoscopic surgery with the tactile feedback and safety of open surgery. Several studies have demonstrated the feasibility and safety of HALDN, making it a preferred approach in many centers.^12^,^13^ In this study, the aim was to present the results of living donor nephrectomies performed at the center using the HALDN technique.

## Material and Methods

Between September 2009 and August 2021, 1221 living donor nephrectomy operations performed by the team at Medicana İstanbul Hospital were retrospectively analyzed. Donor characteristics such as age, gender, body mass index (BMI), donor groups (spouse, relative, ethics committee-approved), surgical side (right/left), type of operation (hand-assisted laparoscopy, conversion from laparoscopy to open surgery, open surgery), operation time, warm and cold ischemia times, amount of bleeding, length of hospital stay, and complications were recorded.

This study was conducted in compliance with ethical standards and approved by the Ethics Committee of Biruni University. The decision/protocol number for the ethics approval is 2024-BİAEK/04-01 dated 21.10.24. Informed consent was obtained from all participants involved in the study prior to data collection.

Data analysis was performed using SPSS version 22.0 (IBM SPSS Corp.; Armonk, NY, USA) software. The normality of continuous variables was assessed with the Kolmogorov–Smirnov test. Normally distributed data were expressed as mean ± SD. Categorical variables were expressed as numbers and percentages.

### Surgical Technique

Donors were placed on the operating table in a 90-degree lateral decubitus position under general anesthesia. A 6-8 cm transverse incision was made below the umbilicus to insert the hand port (Endopath Dextrus, Ethicon), and intra-abdominal pressure was set to 10-12 mm Hg using CO_2_ insufflation. The surgeon inserted their non-dominant hand into the abdomen through the port. A 15 mm trocar was placed 4-6 cm medial to the anterior superior iliac spine, an 11 mm trocar was placed in the upper quadrant from the midclavicular line, and a 5 mm trocar was inserted anterior to the 12th rib. The operation was initiated using a 30-degree angled laparoscope. The colon was mobilized medially to expose the kidney, followed by hilum dissection and preparation of the renal artery and vein. The kidney was extracted through the hand port and taken to the back table. A 10 mm Jackson-Pratt drain was placed in the surgical area for all patients.

## Results

Upon examining the demographic data, donors ranged in age from 19 to 87 years, with a mean age of 48.50 ± 12.75 years. Of the donors, 54.8% were female (n = 669) and 45.2% were male (n = 552). Body mass index ranged from 16 to 41.09 kg/m^2^, with an average of 27.99 ± 3.7 kg/m^2^. Among donor groups, spouse donors constituted 21.4% (n = 261), relative donors 73% (n = 891), and ethics committee-approved donors 5.6% (n = 69) (Table 1). When surgical data were evaluated, 21.5% (n = 263) of the total 1221 living donor nephrectomy operations were performed on the right side, and 78.5% (n = 938) on the left side. In right donor nephrectomies, open surgery was performed in 29.3% (n = 77), conversion from laparoscopy to open surgery in 8% (n = 21), and hand-assisted laparoscopic surgery in 62.7% (n = 165). In left donor nephrectomies, open surgery was performed in 1.8% (n = 17), conversion from laparoscopy to open surgery in 3.5% (n = 33), and hand-assisted laparoscopic surgery in 94.7% (n = 888) (Table 2). The mean operation time was 108.62 ± 29.86 minutes, with no statistically significant difference in operation time between right and left nephrectomies (*P* > .05). Warm ischemia times ranged from 45 to 240 seconds, with a mean of 99.21 ± 56.67 seconds. Cold ischemia times ranged from 33 to 90 minutes, with a mean of 53 minutes and 50 seconds (Table 3). The amount of bleeding ranged from 10 to 700 mL, with an average blood loss of 70 mL. Hospital stays varied between 3 and 12 days, with an average stay of 4.12 ± 1.2 days (Table 4).

When complications were examined, postoperative atelectasis was observed in 7 patients (0.6%). Conversion from laparoscopy to open surgery due to bleeding occurred in 40 patients (3.3%), and 4 patients (0.3%) required blood transfusions. Two patients (0.16%) required laparotomy due to prolonged ileus. Incisional hernia developed in 4 patients (0.33%); all of these patients had a BMI >30 kg/m^2^. Wound infection was detected in 2 patients (0.16%), and scrotal swelling in 3 patients (0.25%). There was no statistically significant difference in complication rates between genders (*P* > .05) (Table 5).

## Discussion

In this study, the outcomes of the HALDN procedures were evaluated and determined that the procedure is a safe and effective method. The mean operation time (108.62 ± 29.86 minutes) and warm ischemia time (99.21 ± 56.67 seconds) are consistent with similar studies reported in the literature.^10^,^11^,^14^ For instance, Ratner et al^10^ reported an average operation time of 231 minutes in their initial applications, but over time, these durations decreased as experience increased. The results indicate that surgeries can be performed in reasonable times thanks to the surgical team’s experience and the standardization of the procedure.

One of the most significant advantages of HALDN is the shorter recovery times and lower complication rates provided by the minimally invasive approach.^8^,^9^,^15^ In this study, the average hospital stay was 4.12 ± 1.2 days, which is consistent with the 3-5 day hospital stays reported in the literature.^16^,^17^ This demonstrates that the laparoscopic approach contributes to faster postoperative recovery for donors.

The most common complications encountered during the operation include bleeding and conversion from laparoscopy to open surgery. In this study, the rate of conversion to open surgery due to bleeding was found to be 3.3%. In the literature, this rate ranges between 1% and 5%.^18^^,19^ This finding suggests that bleeding is a manageable complication in the HALDN procedure and that conversion to open surgery is a safe option when necessary.

The increased risk of incisional hernia in patients with a BMI >30 kg/m^2^ highlights the impact of obesity on surgical complications. The fact that all 4 patients who developed incisional hernia had a BMI over 30 kg/m^2^ indicates that surgical techniques need to be applied more carefully in this patient group. The literature also states that the risk of incisional hernia increases in obese patients and that special attention should be given to closing port sites.^20^

The wide age range of donors (19-87 years) and the acceptable average BMI values (27.99 ± 3.7 kg/m^2^) demonstrate the applicability of HALDN in a broad patient population. Although surgical risks may be thought to increase in elderly donors, no significant complication rates were detected in the older age group in this study. This shows that HALDN can be safely performed when appropriate patient selection is made.^21^

The absence of a significant difference in complication rates between genders indicates that the method is safe for both male and female donors. The literature also reports that gender does not have a significant effect on complication rates.^22^

The limitations of this study include its retrospective nature and reflection of a single-center experience. However, the data obtained from a large patient population and a long follow-up period provide important information on the safety and efficacy of HALDN.

The HALDN method can be considered a safe, effective, and minimally invasive alternative for donor nephrectomy. The advantages provided by minimally invasive surgery make it possible for donors to experience less postoperative pain, recover faster, and return to their normal lives more quickly. This can encourage organ donation and play an important role in reducing the number of patients waiting for organs.

## Figures and Tables

**Table 1.** Demographic Characteristics of Donors

Variable	Value
Age (mean ± SD)	48.50 ± 12.75 years (range: 19-87 years)
Gender	Female: 669 (54.8%)
	Male: 552 (45.2%)
Body mass index (BMI)	27.99 ± 3.7 kg/m^2^ (range: 16-41.09 kg/m^2^)
Donor groups	Spouse: 261 (21.4%)
	Relative: 891 (73%)
	Ethics Committee Approved: 69 (5.6%)

This table shows the distribution of donors' age, gender, BMI, and donor groups.

**Table 2.** Surgical Data and Methods Applied

Surgical Side	Total Number	Open Surgery	Conversion to Open Surgery	Hand-Assisted Laparoscopy
Right donor nephrectomy	263	77 (29.3%)	21 (8%)	165 (62.7%)
Left donor nephrectomy	938	17 (1.8%)	33 (3.5%)	888 (94.7%)

This table presents the distribution of donor nephrectomies by surgical side and the surgical methods used.

**Table 3.** Operation and Ischemia Times

Variable	Value
Operation time	108.62 ± 29.86 minutes
Warm ischemia time	99.21 ± 56.67 seconds (range: 45-240 seconds)
Cold ischemia time	53 minutes 50 seconds (range: 33-90 minutes)

This table shows the operation duration along with warm and cold ischemia times.

**Table 4.** Amount of Bleeding and Length of Hospital Stay

Variable	Value
Amount of bleeding	Average 70 mL (range: 10-700 mL)
Length of hospital stay	4.12 ± 1.2 days (range: 3-12 days)

This table provides information on intraoperative blood loss and the postoperative hospital stay duration for patients.

**Table 5.** Postoperative Complications

Complication	Number (n)	Percentage (%)
Postoperative atelectasis	7	0.6
Bleeding	44	3.6
Conversion to open surgery	40	3.3
Blood transfusion required	4	0.3
Prolonged ileus (requiring laparotomy)	2	0.16
Incisional hernia	4	0.33
Wound infection	2	0.16
Scrotal swelling	3	0.25

This table lists the postoperative complications observed and their frequencies. All patients who developed incisional hernia had a BMI > 30 kg/m^2^. There was no statistically significant difference in complication rates between genders (*P* > .05).

## Data Availability

The data supporting the findings of this study are available from the corresponding author upon reasonable request. Any release of data will comply with ethical and institutional guidelines to ensure the privacy and confidentiality of study participants.
